# Metachronous Synovial Sarcoma After Treatment of Mixed Germ Cell Tumor in a Child with Complete Gonadal Dysgenesis

**DOI:** 10.4274/jcrpe.4905

**Published:** 2018-02-26

**Authors:** Feryal Karahan, Elvan Çağlar Çıtak, Emel Yaman, Mehmet Alakaya, Fatih Sağcan, Eda Bengi Yılmaz, Funda Kuş, İclal Gürses, Yüksel Balcı

**Affiliations:** 1 Mersin University Faculty of Medicine, Department of Pediatric Oncology, Mersin, Turkey; 2 Mersin University Faculty of Medicine, Department of Medical Oncology, Mersin, Turkey; 3 Mersin University Faculty of Medicine, Department of Pediatrics, Mersin, Turkey; 4 Mersin University Faculty of Medicine, Department of Radiation Oncology, Mersin, Turkey; 5 Mersin University Faculty of Medicine, Department of Pathology, Mersin, Turkey; 6 Mersin University Faculty of Medicine, Department of Radiology, Mersin, Turkey

**Keywords:** Gonadal dysgenesis, synovial sarcoma, dysgerminoma, gonadoblastoma, embryonal carcinoma

## Abstract

Patients with complete XY gonadal dysgenesis (GD) show a high predisposition to germ cell tumors (GCT). Patients with coexistence of GCT and GD have been reported previously. Here we present a 15-year-old girl with mixed GCT and GD who also developed an intra-abdominal synovial sarcoma one year after the treatment. This is the first report, to our knowledge, of synovial sarcoma associated with XY GD.

## What is already known on this topic?

Complete 46,XY gonadal dysgenesis (GD) patients show a high predisposition to germ cell tumors. Gonadoblastomas and dysgerminomas are the most frequent histotypes.

## 

### What this study adds?

This is the first report of synovial sarcoma in patients with GD.

## Introduction

A complete 46,XY gonadal dysgenesis (GD) syndrome, known as Swyer syndrome, is characterized by a female phenotype with bilateral streak gonads, normal, female, external genitalia, presence of Müllerian duct and deficient secondary sexual characteristic development with primary amenorrhea (1). Patients with Swyer syndrome show a high predisposition to ovarian cancer. The most frequent observed histotypes are gonadoblastomas and dysgerminomas, followed by Brenner tumors, malignant teratomas and mixed endodermal sinus tumors (2). The lifetime risk of gonadal tumors is in the range of 15-35% in patients with GD (3,4). Germ cell tumors (GCT) were reported in patients with GD but not in conjunction with other types of malignancy. In this report we present a 15 years-old girl patient with mixed GCT and GD who underwent a metachronous somatic malignant transformation (SMT) resulting in intra-abdominal synovial sarcoma one year after the treatment.

## Case Report

A 15-year old girl was admitted to our hospital with a complaint of abdominal distension. Her abdominal examination revealed the presence of a large pelvic mass. Menstrual history revealed that she had never attained menarche. Her height was 171 cm [+1.95 standard deviation score (SDS)], and her weight was 73 kg (+2.98 SDS). Her body mass index was 25 kg/m^2^. Systemic examination showed normal female external genitalia, a “rough” voice, small breasts and a hypoplastic vagina.

**Figure 1:**
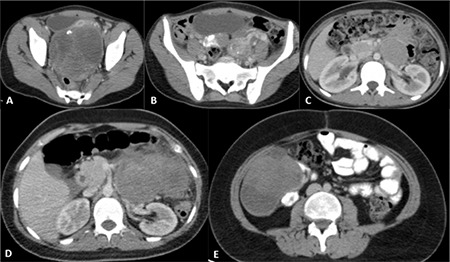
A huge mass with internal cystic components and calcifications, within the cul de sac. Note that the bladder and uterus are displaced anteriorly and the rectum is displaced posteriorly by the lesion (A). Another lesion with calcifications. A small cyst is also visible at the upper level (B), retroperitoneal lymphadenopathy with mild compression on the left renal vein (C). Axial computed tomography image at the renal sinus level shows a huge mass with irregular borders and heterogeneous enhancement within the left retroperitoneal localization, anterior to the left kidney (D). A hypo dense mass with mild irregular contours in the right abdomen at the posterolateral aspect of the caecum and ascending colon (E)

Initial hormonal assays showed elevated levels of serum follicle stimulating hormone at 54.3 IU/mL (3.5-12.5) and luteinizing hormone at 50.82 IU/mL (2.4-12.6). Other endocrinological evaluations were as follows: progesterone 0.706 ng/mL (0.4-1.4); estradiol 45 pg/mL (13-71); testosterone 0.26 ng/mL (8-80); DHEA-SO4 327 µg/dL (65-368); and  androstenodione 2.02 ng/mL (0.5-4.8). On contrast-enhanced, computed tomography (CT), there was a mass, separate from the uterus, which was 12 cm in diameter and which filled the rectouterine space. The mass was mostly multicystic, but also contained solid areas and coarse calcifications (Figure 1A). Lateral to this lesion, there was another solid mass with a diameter of 5 cm and internal calcifications (Figure 1B). In the left para-aortic region, there were two soft tissue lesions adjacent to each other, with dimensions of 10 cm and 3 cm (Figure 1C). The attenuation feature of the larger lesion was similar to that of the lesion in the recto-uterine space, while the smaller appeared to be solid. By radiological appearance, the masses were evaluated as a bilateral malignant ovarian tumor with lymphatic metastases. Laboratory tests revealed high levels of serum a-fetoprotein (AFP) (19931 IU/mL) and Cancer Antigen 125 (Ca 125) (566.3 U/mL) with normal lactic acid dehydrogenase and beta human chorionic gonadotropin (b-hCG) levels. Cytogenetic studies revealed a 46 XY genotype. No germ line deletion or translocation of the sex-determining region Y (*SRY*) gene was detected by fluorescence *in situ* hybridization. Bilateral gonadectomy, Müllerian duct extraction and tumor resection were performed. Pathological investigation showed dysgerminoma (90%), embryonal carcinoma (7%) and gonadoblastoma (3%) on the left side, a dysgenetic gonad and pure gonadoblastoma on the right side (Figure 1D,E,F). Adjuvant chemotherapy was performed with six cycles of cisplatin/etoposide/bleomycin. The patient was in remission after chemotherapy.

**Figure 2:**
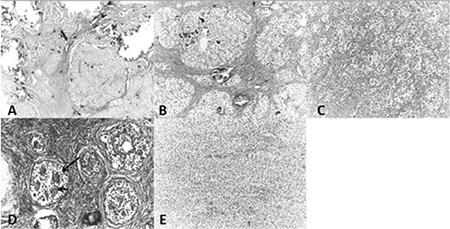
The growth of the gonadoblastoma occurred as rounded nests separated by fibrous stroma that contained significant calcification (hematoxylen and eosin, x40) (A). The germ cells, similar to dysgerminoma cells, in the gonadoblastoma (hematoxylen and eosin, x200) (B). Dysgerminoma. Nests of dysgerminoma cells were separated by fibrous septa containing lymphocytes and plasma cells. The tumor cells had round vesicular nuclei with prominent nucleoli and abundant pale cytoplasm (hematoxylen and eosin, x400) (C). Gonadoblastoma was detected within the streak ovary. The tumor contained a nest of predominantly sex cord-like cells distributed around hyalinized acini (arrow) (hematoxylen and eosin, x400) (D). Synovial sarcoma (monophasic component). Sheets of uniform small spindle cells with ovoid nuclei and scanty cytoplasm (hematoxylen and eosin, x200) (E)

Eighteen months later she was admitted to the emergency department with abdominal pain and distension. A left abdominal mass was detected on physical examination. Abdomino-pelvic CT showed a huge mass with irregular borders and heterogeneous enhancement within the left retroperitoneal region, anterior to the left kidney (Figure 2A). Serum AFP, Ca125, and b-hCG levels were within the respective normal ranges. The mass was near totally resected. Pathological investigation showed a monophasic synovial sarcoma (Figure 2B). Ifosfamide, mesna and doxorubicin chemotherapy protocol was given for six cycles and radiotherapy was added at a dose of 45 Gy. Twelve months later the patient had a further relapse on the right side of the abdomen. Abdominal CT showed a hypodense mass, with mild irregular contours at the posterolateral area of the caecum and ascending colon (Figure 2C). The tumor was partially resected. Ifosfamide/Carboplatin/Etoposide chemotherapy was initiated. Forty-five months after initial diagnosis and 27 months after diagnosis of the synovial sarcoma, the patient died due to resistant/progressive disease.

## Discussion

Patients with GD are phenotypically female with unambiguously female genital appearance at birth and with normal Müllerian structures. The condition typically presents at an age when puberty would be normally expected with primary amenorrhoea and delayed puberty. Although the etiology is not completely understood, 46,XY GD results from failure of testicular development due to disruption of the underlying genetic pathways and several genes, including *SRY* (gene deletion or loss of function mutations; Yp11.3), *NR5A1* (9q33) and *DHH* (homozygous or compound heterozygous mutations; 12q13.1) have been implicated. In addition, patients with partial duplications of Xp (including the *NR0B1* gene) and chromosome 9p deletions (involving the *DMRT1* and *DMRT2* genes) may also present with isolated 46,XY complete gonadal dysgenesis (CGD). Rarely mutations in the CBX2 gene and also dublication of DAX1 gene have been  considered responsible for the development of 46, XY CGD(5,6,7). Mutations in the *MAP3K1 *gene (located on chromosome 5q) that cause downstream alterations in the MAP kinase signaling pathway have been identified (8).

Patients with GD have a 30% risk of development of gonadoblastoma with a 50-60% risk of malignant transformation, typically to dysgerminoma (9). The risk of malignancy in patients with GD increases with age; it has been reported that the risk is 50-70% in the third decade and as high as 80% in the fourth (10). In patients with primary amenorrhoea, GD should always be kept in mind due to the high risk of malignant transformation. Bilateral gonadectomy is advised as soon as the diagnosis is made (11). Unfortunately, our patient had bilateral gonadectomy and Müllerian duct extractions only after the disease had progressed to bilateral GCT.

Gonadoblastomas are rare, mixed, germ cell, sex cord, stromal tumors, almost exclusively seen in patients with underlying gonadal disorders. Germ cells in dysgenetic gonads are genetically unstable and tumorigenic because patients with GD have a higher risk of development of germ cell tumor. It has been hypothesized that the Y chromosome contains a gonadoblastoma locus, responsible for this benign tumor, that may also develop bilaterally and coexist with other neoplasms, such as dysgerminoma (12). Radaković et al (13) reported that 55% of patients suffering from GD were diagnosed with gonadoblastoma or dysgerminoma. Cases of co-occurrence of gonadoblastoma with dysgerminoma and gonadoblastoma with choriocarcinoma have also been reported (12). It is assumed that gonadoblastomas are unstable and may result in choriocarcinoma (14). Our patient had a pure gonadoblastoma in her right ovary and dysgerminoma, embryonal carcinoma and gonadoblastoma on the left side.

GCTs include a diverse group of tumors that arise from primordial germ cells, either in the gonads or in non-gonadal sites. SMT of a GCT means the occurrence of somatic non-germ cell malignancy. Faure Conter et al (15) reported that SMT associated with GCT in children is rare and that these are aggressive tumors with various primary lesions, various GCT histologic subtypes, and poor overall prognosis. Although the presentation is mostly synchronous in children, most of the adult cases are metachronous. Giannatempo et al (16) reported that the median delay between SMT and GCT was four years with a maximum of 18 years. They also reported that SMT occurred concurrently with GCT in only 40% of patients. Different studies showed that 78.5-100% of SMT were diagnosed at the same time as the GCT diagnosis. Different malignant tumors such as rhabdomyosarcoma, peripheral primitive neuroectodermal tumors, adenocarcinoma, squamous cell carcinoma, osteosarcoma, angiosarcoma, leukemia, neurosarcoma, undifferentiated sarcoma, myxoid sarcoma, fusiform cell sarcoma, bronchoalveolar sarcoma and thyroid carcinoma have been reported to arise in GCT (17). Our patient had a metachronous presentation, similar to that reported in adult cases, after 18 months from the initial diagnosis and she had no GCT at that time. Tumor markers were negative at the diagnosis of synovial sarcoma.

In most of the reported cases with SMT, primary diagnoses were teratoma (15,16,18). GCTs have a capacity to display totipotential differentiation. Some authors explain this condition with malignant transformation of the yolk sac or with teratoma cells while others propose a divergent differentiation of primordial stem cells toward GCT and SMT (15,16,18,19). Specific chromosomal changes trigger this transformation. The presence of isochromosome 12p, a specific marker of GCT and of chromosomal abnormalities in MT of non-GCT, strongly favor this hypothesis. We did not investigate the presence of isochromosome 12p in our patient. It is possible that this chromosomal aberration may have caused the metachronous tumor development. The patient received cisplatin/etoposide/bleomycin for the treatment of GCT. The most well known late complications of etoposide are dose related myelodysplastic syndrome and secondary acute myeloid leukemia (20,21,22). A similar complication (leukemia) is reported for cisplatine in many studies (23,24). The most important late complication of bleomycin is pulmonary toxicity. These complications, especially the secondary cancers usually occur five years after treatment. In our patient, synovial sarcoma occurred 18 months after the treatment and we believe that the tumor developed metachronously, rather than as a complication of the chemotherapeutics given in this case.

We could find no other case reports of patients with GD developing synovial sarcoma after GCT. Thus, to the best of our knowledge, this is the first report of GD with synovial sarcoma following GCT.

Bilateral gonadectomy and Müllerian duct extractions have to be considered for newly diagnosed patients with GD as a risk reducing strategy for development of malignancy. Patients with GCT and with chromosomal or genetic defects must be carefully followed and observed because of the high risk for development of synchronous or metachronous SMT.
